# Pneumoblastoma: A Rare Tumor in Children Revealed by Pyopneumothorax

**DOI:** 10.7759/cureus.17539

**Published:** 2021-08-29

**Authors:** Germaine Niyitanga, Ibtissam Zouita, Dounia Basraoui, Hicham Jalal

**Affiliations:** 1 Department of Radiology, University Hospital Mohammed VI, Marrakesh, MAR

**Keywords:** pneumoblastoma, lung tumor, malignant, child, imaging

## Abstract

Pneumoblastoma is a rare but severely aggressive primary lung tumor. Exceptional at the pediatric age, pneumoblastoma lacks clinical and radiological specificity. It is rarely mentioned as a first-line differential, as radiological images are often confused with those of congenital lung malformation. This history is reminiscent of the diagnostic difficulty with which the clinician is confronted with the finding of an intrathoracic cystic image of the child. Primary childhood lung tumors, especially pneumoblastoma, are difficult to diagnose both clinically and by imaging. It is necessary to think about it in the face of any lingering respiratory infection and any atypical radiological presentation. We report this case in order to illustrate the usual radiographic, ultrasound, and scenographic aspects of this rare malignant tumor in children and to highlight the diagnostic problems posed by this exceptional pathology.

## Introduction

Pneumoblastoma is a rare but severely aggressive primary lung tumor. It accounts for about 0.5% of primary malignant lung tumors. The initial tumor is localized on the periphery of the lungs and can reach the pleura [1]. Pneumoblastoma lacks clinical and radiological specificity. It is rarely mentioned as primary diagnosis, as radiological images are often confused with those of a congenital pulmonary malformation. Through this case, we highlight the diagnostic problems posed by this pathology and propose to review the literature of the usual radiological aspects of this rare malignant tumor in children.

## Case presentation

**Figure 1:**
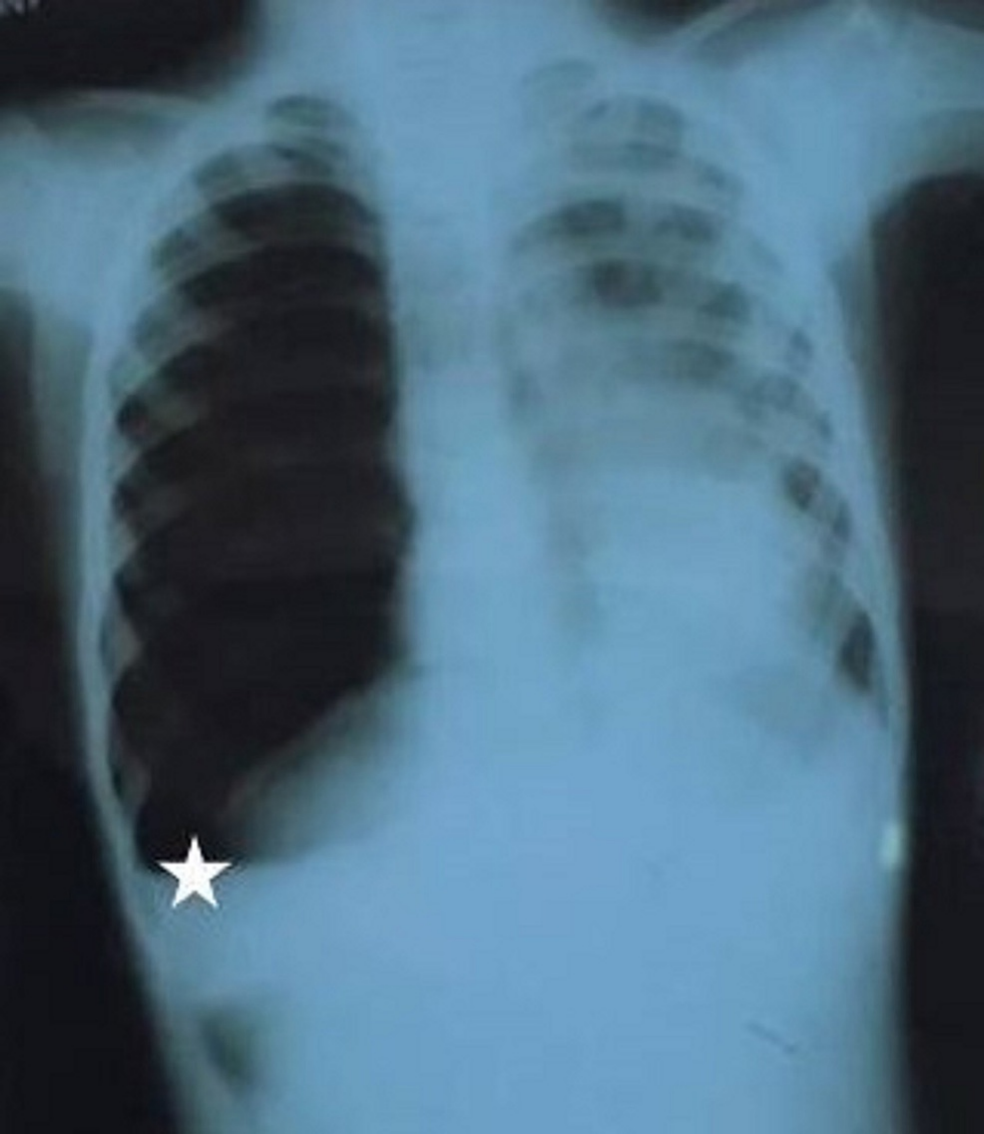
Standard chest x-ray Chest x-ray showing the presence of a right hydropneumothorax.

We report a case of pneumoblastoma occurring in a two-year-old girl who showed up with an emetic cough complicated by respiratory distress. The standard chest x-ray showed a right hydropneumothorax (Figure 1).

**Figure 2:**
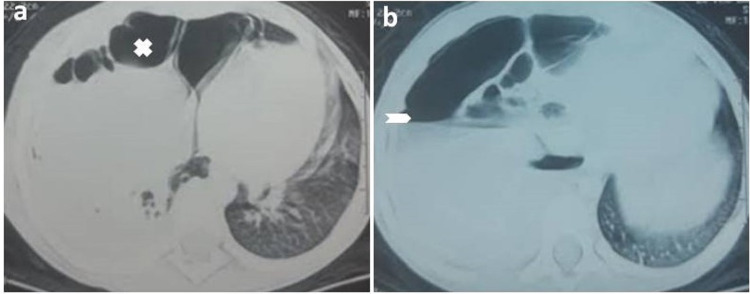
Thoracic CT scan Axial CT scan showing overdistension of the right chest with cystic air-containing lesions (a) with significant hydropneumothorax (b) diagnosed preoperatively as CCAM. CCAM: congenital cystic adenomatoid malformation.

Chest ultrasound revealed a large right pleural effusion with finely echogenic content, containing air bubbles, which turned out to be pyopneumothorax after pleural puncture. Bacteriological examination of pus isolated *Pseudomonas aeruginosa* and *Klebsiella pneumoniae*. Other laboratory data showed leukocytosis at 16,500/mm^3^, predominantly neutrophilic at 8420/mm^3^, hemoglobin at 8 g/dl, and C-reactive protein (CRP) at 80 mg/dl, and the blood culture was sterile. The patient was put on antibiotic therapy (amoxicillin-clavulanic acid) without improvement. Following this, a thoracic computed tomography was ordered and showed cystic air-containing lesions with significant hydropneumothorax suggesting a bronchopulmonary malformation such as congenital cystic adenomatoid malformation (CCAM) (Figure 2).

**Figure 3:**
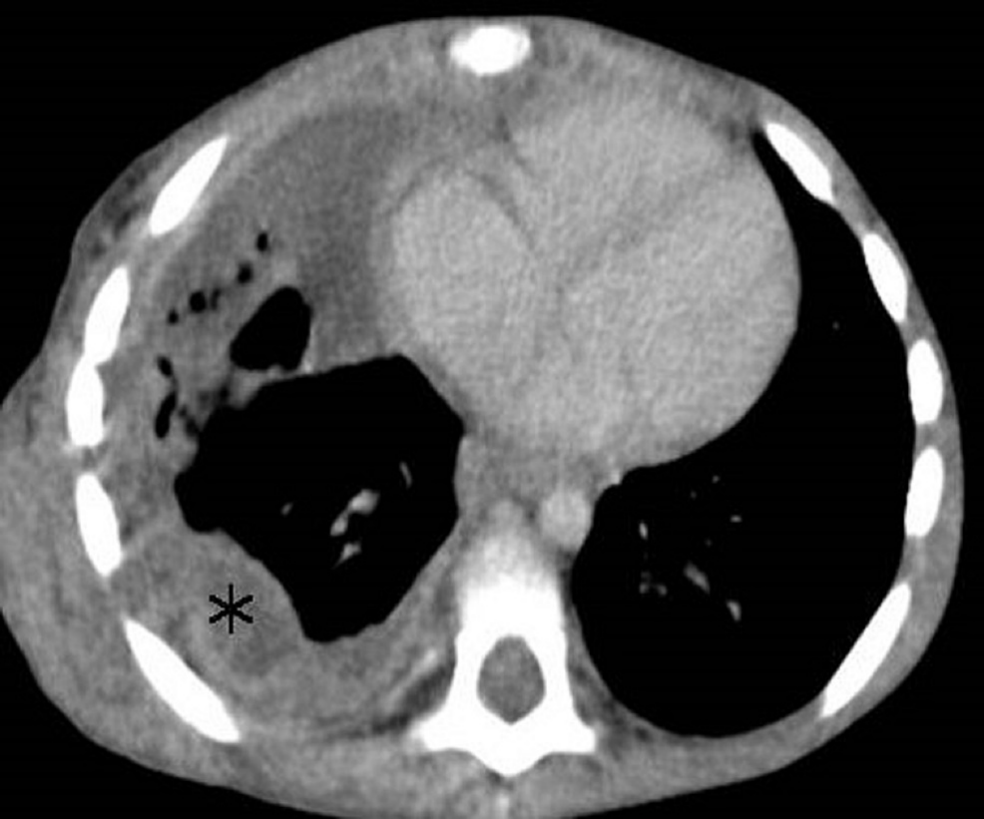
Thoracic CT scan Axial CT showing an irregular posterior and right lateral basal pleural (star) thickening suggestive of a recurrence.

**Figure 4:**
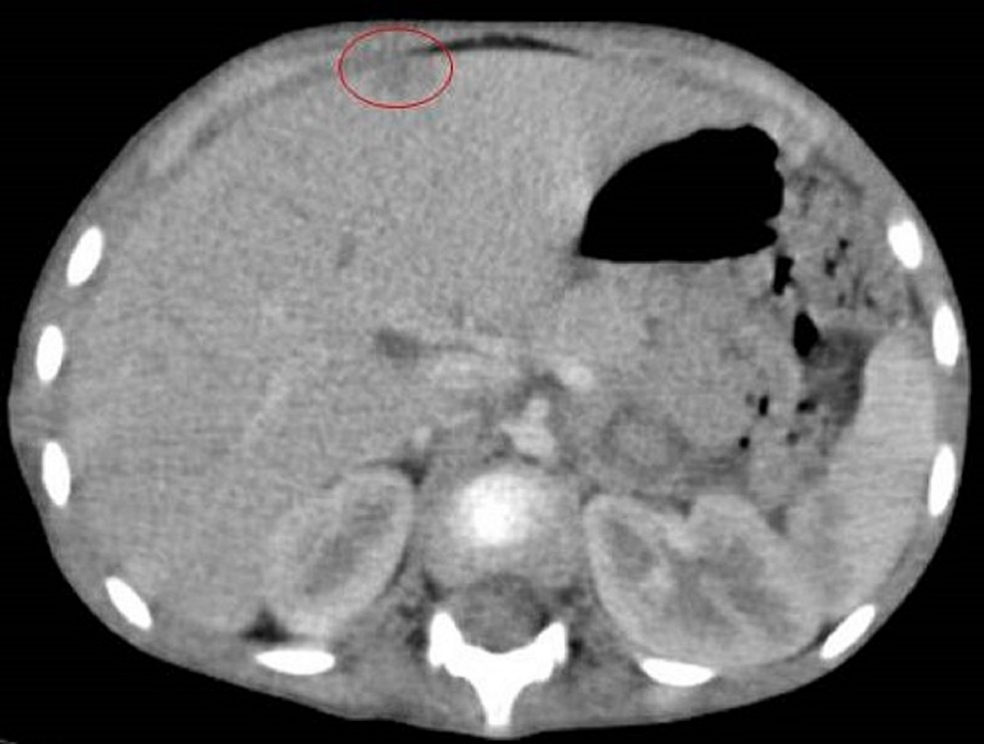
Thoracic CT scan CT scan showing a nodular hepatic lesion astride segments III and IV of secondary origin (circle).

The patient underwent surgical resection and the histology confirmed the diagnosis of high-grade pneumoblastoma. On the extension workup, the cerebral CT scan was normal while the thoraco-abdominopelvic CT scan revealed an irregular posterior and right lateral basal pleural thickening suggestive of a recurrence (Figure 3), associated with ipsilateral pulmonary nodules as well as a nodular hepatic lesion astride segments III and IV of secondary origin (Figure 4). Adjuvant chemotherapy has been indicated in addition to surgery.

## Discussion

Pneumoblastoma is a rare intrathoracic tumor that occurs among children under five years of age, as a rule of high malignancy. Macroscopically, three types can be distinguished type I purely cystic, bullous; type II combines solid and cystic plaques, and type III which is exclusively solid [2]. The pulmonary tumor pathology of the child is mainly represented by pulmonary metastases while primary malignant lung tumors are extremely rare [2]. Pneumoblastoma, a primary lung tumor, is the third common primary malignant tumor in children and accounts for 0.5% of primary lung malignancies [1]. It occurs almost exclusively in children under the age of 15, with a peak in frequency between two and four years, without gender predominance. In addition, it is a tumor that can be found in all races [3].

The positive diagnosis of pneumoblastoma is based mainly on histopathology but must take into account a body of anatomo-clinical and radiological arguments. Its clinical expression is not very specific. The respiratory symptoms most frequently found are symptoms such as cough, chest pain, recurrent pneumonia, or even hemoptysis. In the majority of cases, the onset of the clinical picture is gradual. This symptomatology is without specificity, being able to go from a simple cough to real respiratory distress with frequently a notion of trailing pneumonia, which is the case in our patient [4,5]. The discovery can be fortuitous in the face of asymptomatic cases.

The standard chest x-ray is the first-line examination for any pulmonary symptomatology. It reveals the existence of the tumor, as it can also follow its evolution in the event of surgical abstention, and finally detect local recurrences after surgical excision. It allows us to study the location and appearance of the tumor [6]. Usually, it presents as a large, single, peripheral lung mass, completely opacifying the hemithorax with mediastinal deviation, which poses a problem of differential diagnosis with parietal or pleural tumors [7]. Rarely, it presents in the form of a pneumothorax or hydropneumothorax as some authors testify, which agrees with our case [8]. Then comes the thoracic ultrasound coupled with Doppler and which generally shows a heterogeneous tissue lesion process, thus making it possible to exclude a pleural effusion in case of an opaque lung on the standard x-ray and to contraindicate the puncture [9]. In our case, on chest ultrasound, pulmonary opacity did correspond to mixed pleural effusion, which turned out to be pyopneumothorax after pleural puncture.

Thoracic computed tomography is the examination of choice to characterize the tumor; it usually takes on the appearance of a hypodense mass, of heterogeneous tissue density, enhanced by the contrast product, the site of readily hypodense areas, which may correspond to necrosis [10]. It can be purely cystic (type I) with congenital cystic adenomatoid malformation (CCAM) type IV as the main differential diagnosis, especially since a good number of authors have demonstrated an association between pneumoblastoma and pre-existing cystic pulmonary lesions, in particular, the adenomatoid malformation cystic [11,12]. However, unusual presentations of pneumoblastoma with pleural effusion and/or pneumothorax/hydropneumothorax have been reported, and Paupe et al. state that pneumothorax is due to rupture of cysts [8,13]. In our case, it was an air-containing cystic structure associated with a hydropneumothorax suggesting a CCAM.

The scanner also makes it possible to carry out a precise extension assessment and finally to rule out differential diagnoses in its purely cystic form, with other pulmonary cystic lesions, namely CCAM, congenital lobar emphysema, pulmonary sequestration, or bronchogenic cyst; or when the lesion presents as a lump or a single pulmonary nodule, with a primary malignant lung tumor (rhabdomyosarcoma) or secondary, a benign lung tumor such as a hamartoma, a chondroma or a sclerosing hemangioma; or when it presents as an endobronchial tumor, with a carcinoid tumor, adenoid cystic carcinoma or mucoepidermoid carcinoma [10].

The indication for magnetic resonance imaging is still very limited. Overall, no radiological character is specific to pneumoblastoma, it can take on various atypical aspects (which is our case) and certain signs can be retained as orientation elements such as the large volume of the tumor, its rapid increase between two successive radiological assessments, essentially peripheral involvement, and necrosis with the presence of intratumoral air [6]. Pneumoblastoma can only be diagnosed through histological examination. Treatment consists of a large surgical resection of the tumor, followed by adjuvant therapy with chemotherapy, possibly combined with radiation therapy.

## Conclusions

Primary lung tumors in children, particularly pneumoblastoma, are rare and difficult to diagnose both clinically and by imaging. It is necessary to think about it when faced with any atypical aspect and any lingering respiratory infection. Imaging guides and reinforces the diagnosis, but confirmation remains histopathological.
